# Interactions between Diabetes and the Heart

**DOI:** 10.1155/2016/8032517

**Published:** 2016-01-24

**Authors:** John Skoularigis, Andreas Melidonis, Dirk Westermann, Vasiliki V. Georgiopoulou, Georgios Karagiannis, Gregory Giamouzis

**Affiliations:** ^1^Cardiology Department, Larissa University Hospital, 41110 Larissa, Greece; ^2^Department of Internal Medicine, Tzaneio Hospital, Athens, Greece; ^3^Department of General and Interventional Cardiology, University of Eppendorf, Hamburg, Germany; ^4^Division of Cardiology, Emory University Hospital, Atlanta, GA, USA; ^5^Transplant Department, Harefield Hospital, London, UK

Diabetes is a chronic metabolic disease that has reached epidemic proportions, affecting millions of individuals worldwide, and the global prevalence of the disease is expected to increase exponentially. Diabetes is associated with substantial mortality, morbidity, and healthcare expenditure.

Cardiovascular complications are the major cause of mortality and morbidity in individuals with diabetes and the largest contributor to the overall cost of diabetes. More than 60% of deaths in patients with diabetes have an underlying cardiovascular cause. Patients with diabetes have 2- to 4-fold higher risk of developing cardiovascular disease than individuals without diabetes [1]. Atherosclerosis has long been recognized as the main causative factor for cardiovascular events in patients with diabetes. Emerging evidence suggests that changes in circulating blood (e.g., inflammatory mediators, altered platelet function, hypercoagulability, hypofibrinolysis, and microparticles) and myocardial factors (e.g., altered metabolic state and neural and vascular impairment) may also play equally important roles in the pathophysiologic process underlying the increased propensity for cardiovascular disease in individuals with diabetes.

In this special issue, we have invited a few papers that address such issues.

*Heart Failure and Diabetes*. People with diabetes have an increased risk of developing heart failure, both as a consequence of coronary artery disease and as a result of cardiomyopathy, which may be due to a microangiopathy or primary cardiomyocyte dysfunction. Indeed, diastolic left ventricular dysfunction is a consistent observation through all levels of glucose intolerance [2]. Dr. K. Hensel et al. investigated whether speckle tracking echocardiography can be used to detect subclinical alterations of left ventricular myocardial deformation in asymptomatic pediatric patients with uncomplicated T1DM. Furthermore, they combined speckle tracking echocardiography with physical stress testing in order to unmask subtle changes of cardiac contractility that might potentially be occult at rest (for and against the existence of diabetic cardiomyopathy). With their results they provide further evidence for diabetes-associated nonischemic cardiomyopathy in T1DM patients as they describe the early stage of diabetic cardiomyopathy which is initiated by hyperglycemia, has insignificant changes in myocardial structure (normal left ventricular dimensions, wall thickness, and mass), has possible substructural changes in myocytes, and can only be detected by sensitive methods such as strain, strain rate, and myocardial tissue velocity. In another study Dr. L. León et al. discuss the current scientific evidences to propose circulating micro-RNA as promising biomarkers for early detection of diabetic cardiomyopathy and then to identify patients at high risk of diabetic cardiomyopathy development. Moreover, they summarize the research strategies to identify micro-RNA as potential biomarkers, the present limitations, challenges, and future perspectives.

Volatile anesthetics, like sevoflurane, have cardiodepressive effects and may aggravate cardiovascular complications intraoperatively. Preservation of myocardial perfusion during surgery is particularly important in patients with increased risk for perioperative complications, such as diabetes. As sevoflurane's vasodilatory impact may be more abundant in patients with cardiometabolic disease, like T2DM, Dr. C. E. van den Brom et al. investigated the additional effect of sevoflurane anesthesia on myocardial perfusion and function in diet-induced prediabetic rats.

Hyperglycemia is associated with altered myocardial substrate use, a condition that has been hypothesized to contribute to impaired cardiac performance. It is caused to a great extent by change of energy metabolism, which is an additional trigger of functional and structural disorders of heart muscle. In turn, remodeling of the cardiomyocyte membranes with advanced glycation end products and free radical oxidation is essential factor in development of diabetes mellitus. Dr. S. Afanasiev et al. showed that in the experimental conditions induction of DM on the stage of formation of postinfarction remodeling increases adaptive ability of myocardium. It is manifested in inhibition of increase in lipid peroxidation processes activity and maintaining of force-interval reactions of myocardium connected with calcium transport systems of cardiomyocyte sarcoplasmic reticulum.

*Antiplatelet Therapy in Diabetes*. The ADP receptor P2Y12 plays a pivotal role in platelet aggregation [3]. This role is emphasized by the results of clinical trials that demonstrate improvement of long-term clinical outcomes in patients treated with the P2Y12 receptor antagonist clopidogrel. However, a high interindividual variability in platelet response to clopidogrel has been described. The fact that subjects with suboptimal platelet inhibition by clopidogrel are at increased risk of cardiovascular ischemic events represents an alarming clinical problem [4]. T2DM was recently connected with a failure in antiplatelet response to clopidogrel and the presence of high on-treatment platelet reactivity was repeatedly associated with the risk of ischemic adverse events. Patients with T2DM show significantly higher residual platelet reactivity on ADP receptor blocker therapy and are more frequently represented in the group of patients with high on-treatment platelet reactivity. The article by Dr. M. Samoš et al. reviews the current knowledge about possible interactions between T2DM and ADP receptor blockers therapy.

*Heart Rate and Diabetes*. Elevated resting heart rate has been associated with increased risk of all-cause mortality and cardiovascular events in healthy subjects as well as those with preexisting cardiovascular disease including hypertension, acute myocardial infarction, and heart failure or left ventricular dysfunction by numerous epidemiological studies [5]. However, limited data are available in T2DM patients. In an interesting study, Dr. V. Bartáková et al. evaluated whether resting heart rate could be a predictor of major cardiovascular events, progression of diabetic kidney disease, and all-cause mortality in a cohort of T2DM patients.

*Adipose Tissue and Insulin Resistance*. Adipose tissue accumulates both within and around internal organs with potential to negatively alter their biological function. Functional abnormalities of the adipose tissue are linked to inflammation, metabolic dysregulation, vascular dysfunction, impaired angiogenesis, and insulin resistance. Interest has focused on the potential role of epicardial adipose tissue as a modulator of cardiovascular function, owing to its immediate anatomic proximity to the coronary vasculature and myocardium with shared microcirculation [6]. Evidence suggests that epicardial adipose tissue could play a role in the pathogenesis of cardiovascular disease and is also potentially involved in the onset and progression of coronary artery disease. Increased expression of receptor for advanced glycation end products (RAGE) in adipose tissue has been associated with inflammation, adipocyte hypertrophy, and impaired insulin signal. The potential role of RAGE in epicardial adipose tissue has not been explored much. The study by Dr. E. Dozio et al. examined the RAGE expression in epicardial adipose tissue and suggests potential involvement in promoting epicardial adipose tissue dysfunction in coronary artery disease patients.

We hope that the readers of the journal will find the topics as interesting and important as we did.

*John Skoularigis* *John Skoularigis* *Andreas Melidonis* *Andreas Melidonis* *Dirk Westermann* *Dirk Westermann* *Vasiliki V. Georgiopoulou* *Vasiliki V. Georgiopoulou* *Georgios Karagiannis* *Georgios Karagiannis* *Gregory Giamouzis* *Gregory Giamouzis*
